# A Clinical Treatise on the Diseases of the Heart, Preceded by Original Researches on the Anatomy and Physiology of That Organ

**Published:** 1836-10

**Authors:** 


### BRITISH AND FOREIGN

### MEDICAL REVIEW,

FOR OCTOBER, 1836.

### PART FIRST.

### Analytical and Critical Reviews.

### ART. I.

*Traité Clinique des Maladies du Cœur, précédé de Recherches nouvelles sur l'Anatomie et la Physiologie de cet Organe.* Par J. BOUILLAUD, Professeur de Clinique Médicale à la Faculté de Médecine de Paris.—*Paris*, 1835. Deux tomes. 8vo. pp. 534, 632.

*A Clinical Treatise on the Diseases of the Heart, preceded by original Researches on the Anatomy and Physiology of that Organ.* By J. BOUILLAUD, Clinical Professor to the Faculty of Medicine at Paris. —*Paris*, 1835. Two volumes.

IN terminating an article on this work in a former Number, we promised speedily to return to it, and give a detailed analysis of its more strictly practical portion. This promise we now proceed to fulfil.

The part which immediately follows that which last occupied us consists of some general remarks on the diseases of the heart. In these the author examines, in succession, their precise seat and anatomical characters; their diagnostic signs, causes, nature, and classification; their progress, duration, and termination; their prognostics and treatment; and, finally, their complications with each other, as well as with diseases of other organs. Nothing is more rare than disease of the *whole* heart: frequently it occupies but one cavity of the organ, or even but one tissue out of all those which go to form the walls of such cavity. The physiological characters or symptoms of diseases of the heart he considers at large, under the two heads of Local Signs and General Signs; and, in the investigation of the former, agrees with all other good modern observers in considering auscultation and percussion as the most valuable of all our resources, and next to these he places inspection and examination by the touch. He points out very forcibly the error into which many systematic writers on diseases of the heart have fallen, of ascribing to *all* its lesions indifferently certain secondary consequences, which are really attributable only to particular ones. Thus, some have erroneously ascribed to pure and simple hypertrophy of the heart those venous congestions and passive serous effusions which, so far from recognizing for their cause such an increase of action as accompanies a true hypertrophy, depend, on the contrary, on the diminution of the force or moving power of the heart, or on a mechanical obstacle to the course of the blood through its cavities. We must admit, he continues, two principal modes in which secondary morbid phænomena are produced in cases of diseased heart,—namely, one which is altogether physical and mechanical, as in the case of displacement and compression of the lungs by an enlarged heart, or in that of passive congestions in a variety of organs, from induration of the valves and narrowing of the orifices opposing the free transmission of blood; whilst the other principle is purely sympathetic or vital, and is exemplified in the fever depending on inflammation of the various tissues of the heart.

The influence exercised over the circulation in general, and especially over that of certain viscera, (as the lungs, the liver, the spleen, and the brain,) by particular diseases of the heart, is remarkably great; and these secondary or symptomatic affections have too often been mistaken for primary inflammatory diseases of the suffering organs, and occasionally even for those of a merely nervous character. We have thus ourselves known headachs from this cause, of such a degree of intensity and so frequent occurrence as to render life miserable, exist from youth up to middle age, without their real source in disease of the heart having ever once been suspected, even by physicians of the first eminence, at least till the fatal termination was approaching, and the supervention of passive dropsies opened the eyes of the attendants to the true nature of the case.

The sympathetic effects connected with diseased heart vary according to the portion of this organ chiefly implicated. Thus, when the left ventricle is the seat of well-marked hypertrophy, the face is for the most part red and animated, and the eyes are brilliant; there is dizziness and confusion of head, and, towards the conclusion, formidable epistaxis, and occasionally even cerebral hæmorrhage. Whereas, if it be the right ventricle which is so affected, (a much rarer case,) slight spitting of blood, or occasionally even pulmonary apoplexy, ensues. The cause of the difference is obvious. If there be any mechanical obstruction to the circulation through the heart, on which soever side it may be situated, it will necessarily give rise to much disturbance in the arterial, venous, and capillary circulation generally: this will, however, vary much in degree and kind, according as the impediment is situated in the right side or in the left: thus, if it be in the left, the lung will feel the first effects of it, though no doubt it will subsequently extend its influence to the right cavities and to the veins which empty themselves into the right auricle. If the obstacle, on the contrary, be in the right side, the venæ cavæ, the veins of the liver, spleen, brain, face, &c., will be the first to suffer, giving rise to passive congestions of the parts just named. But it is erroneous, however, to state absolutely, as is done by some authors, that the effects just detailed are exclusively connected with affections of the right side; although they certainly manifest themselves much earlier here, and in a higher degree. M. Bouillaud lays much stress on mechanical obstruction as a cause of the above derangements of the circulation than Dr. Hope, though he readily admits that simple dilatation, hypertrophy, &c. may occasionally give rise to them. Testa has placed amongst the incidental consequences of diseases of the heart the destruction of the eyeball, depending probably on deep-seated derangement in the circulation of the capillaries of the part; as also gangrene of the limbs; and in the present work there are one or two cases illustrative of the latter, as it occurs in the advanced period of heart disease: but M. Bouillaud does not look upon either of these occurrences as its direct consequence. In the cases of gangrene, he has usually found a coagulum in the artery leading to the part; and he looks upon this as the immediate cause of the affection.

*Causes.* In his chapter on the Etiology of the diseases of the heart, though he by no means impugns the influence of the special causes to which they are by most writers attributed, he is of opinion that the fact that they may originate, like the disorders of other organs, under the influence of general causes, is too often lost sight of; for he is convinced that in these, and more especially in exposure to cold and the consequent inflammation, we shall find their chief source. The laborious and almost unceasing action of this organ; its sympathy with all the other parts of the body, so strikingly manifested in acute and chronic local affections, accompanied by fever; its liability to suffer from long-continued and violent muscular efforts, and everything which embarrasses the lungs, too stimulant a diet, moral affections, and a host of other causes so eloquently detailed by Corvisart, are facts which need only be mentioned to satisfy us that it is exposed to a greater number of deranging influences than perhaps any other organ in our system.

Corvisart, and still more recently an Italian writer, M. Schina, has dwelt very largely on the influence of the moral affections in the production of diseases of the heart; and we cannot agree with M. Bouillaud when he endeavours to limit their operation to the development of mere nervous disorders of this organ, excluding, in a great degree, from their range those of an acute or of an organic nature. We fully join him, however, in dissenting from Corvisart's assertion, that the heart is placed out of reach of the influence of sudden atmospheric changes; for it is matter of daily observation that pericarditis, for example, originates readily under the same external influences as a pleurisy or an acute rheumatic attack; and this is likewise the case, according to our author, in regard to inflammation of the internal lining membrane of the heart; a disease which so often coexists, as already stated, with pericarditis, and a just knowledge of which, he thinks, is destined to work a complete revolution in respect to our notions on the pathology of the heart.

An hereditary predisposition to diseases of the heart is in some instances unquestionable, and has long since been sufficiently established by Morgagni, Senac, Corvisart, and others. Lancisi, in particular, mentions that a great-grandfather, grandfather, father, and son, were successively affected with aneurism of the heart; Albertini speaks of a woman and her five brothers, all martyrs to the same affection; M. Bouillaud says he has daily proof of the opinion being well founded; and we may add, that our own observation (agreeing, we doubt not, with that of every experienced physician,) enables us to attest the same.

The chief object of M. Bouillaud's book, which seems to be to prove that the great majority of diseases of the heart originate in acute and chronic inflammations of its tissues, is very largely developed in the present section. He asserts that, before he had become possessed of numerous and exact records of cases of this kind, such as his work abounds in, and more especially of endocarditis, or inflammation of the inner lining membrane of the organ, he had but very confused notions on many questions relative to organic diseases of the heart; but that, when once he had obtained a collection of accurate facts bearing on the subject, and made them the object of his careful study, his eyes became as it were unsealed, and a new light burst in on him. That, in a very great majority of cases, the lesions alluded to have really an inflammatory origin, especially when occurring in the more vigorous periods of life, or when they have been obviously preceded, at some time or other, by acute symptoms, must, we think, be fully admitted by all those who take fairly into consideration the structure and pathological analysis of the parts affected.

With regard to the formation of a satisfactory classification of the diseases of the heart, much has been attempted, but little has been done hitherto. Nature conforms herself to no artificial arrangements, and diseases of the heart, even more than those of other organs, present themselves almost invariably in a complex form; two or more morbid conditions for the most part coexisting, and especially if the case offer itself to our observation at an advanced stage.

*Progress.* It is a curious circumstance that the symptoms of organic disease of the heart are, to a certain degree, of an intermittent nature. This was alluded to by Corvisart, who adduces two instances of aneurism of the aorta simulating, from this cause, spasmodic asthma. Some later French pathologists, amongst whom we believe we may place M. Rostan, conceive that all cases of so-called nervous periodic asthma are merely symptomatic of organic affections within the chest. M. Bouillaud, on the other hand, thinks that the periodicity is not really the attribute of the organic disease, but is rather to be ascribed to the coexistence of a neurosis of the heart. However it is to be explained, the fact of the symptoms of heart disease being liable to frequent exacerbations or violent paroxysms is indubitable. These sometimes come on without any known cause; whilst, at others, they may be traced up to some excess in diet or exercise, to mental impressions, or atmospheric changes.

*Prognosis.* In diseases of the heart, the prognosis has lost something of the gloomy characters it possessed in the time of Senac, and even so late as in that of Corvisart. Some of them are, no doubt, essentially and almost immediately mortal; such as rupture of the heart, sudden coagulations of the blood within it, and certain cases of syncope. Amongst those which are in their nature incurable, though not immediately mortal, are to be reckoned induration of the valves with considerable narrowing of the orifices, and certain instances of pericarditis and carditis in the chronic stage. The two last-mentioned diseases, whilst still in the acute stage, though very serious in their nature, are yet, when well treated, by no means so generally mortal as Corvisart supposed; and inflammation of the internal surface of the heart likewise, (an affection which was unknown to that distinguished physician,) though even more dangerous still, if neglected or mismanaged, is amenable to treatment, and in very many cases capable of being completely cured under the influence of active and judicious measures. Hypertrophy of the heart, when unaccompanied by any very considerable dilatation or contraction of its cavities, or serious lesion of its valves; simple thickening of the valves, without any serious obstacle to the course of the blood; and, finally, adhesions, and fibrous or cartilaginous patches in the pericardium, do not enter into the category of diseases necessarily mortal; for, with good management, the individuals labouring under them may have their lives protracted to a very advanced period. The importance of the functions of this organ, and the impossibility of placing it in a state of repose, will always necessarily render diseases of the heart proportionably much more dangerous than those of almost any other organ: still, in a very considerable number of instances, despondency is unwarrantable.

As the diagnosis of heart diseases has of late years made such considerable advances, it was only natural to expect that the treatment should have acquired greater precision, and have become proportionably more successful; and that it should become daily rarer for us to see chlorotic individuals, and others with merely functional derangements of this organ, subjected to bleeding and other prejudicial lowering means, where tonics and a nutritious diet could alone be of service; or the equally deplorable error committed of treating solely with antispasmodics the palpitations and dyspnœa of patients labouring under an organic affection. The peculiarity of M. Bouillaud's method in the treatment of inflammatory affections of the heart, (and we have seen how extensive a range he attributes to these,) consists in a very free use of antiphlogistic measures, ("emissions sanguines à haute dose,") by means of which, he assures us, he has been successful in an eminent and almost unexampled degree; having latterly been able to save almost all the patients labouring under such diseases, when they applied to him in their early stage. In looking to his cases, however, notwithstanding his assumption of originality in his treatment, the bloodlettings described in them do not strike us as being larger than those usually practised in similar cases in this country, ("trois à quatre palettes,"—twelve to sixteen ounces,) though they are performed perhaps at somewhat shorter intervals, and rather longer persevered in. Of the value of this method, within due limits, there cannot, we conceive, be a doubt, as well from its influence in reducing inflammation generally, as from its being the only method of which we are possessed of reducing the quantity of labour to be borne by this organ, and thus placing it in a comparative state of repose. Half measures, as he justly remarks, only suffer the disease to pass into a chronic condition, with all its unmanageable consequences, which tend to convert life into a lengthened and hopeless disease. He quite agrees with Corvisart in the propriety of limiting within very narrow bounds the employment of Valsalva's and Albertini's method in organic affections of the heart. In the efficacy of mercury alone, or in combination with opium, in controlling inflammation of an ordinary character, and especially that of serous membranes, and of limiting the effusion of coagulable lymph, preventing its organization and promoting its absorption, he does not seem to entertain that confidence which has long been so characteristic of British practice, and which is beginning to be felt latterly also in Germany. He is thus deprived of a powerful auxiliary at that period when bloodletting is no longer admissible. He is not ignorant of, or altogether sceptical as to the influence of this remedial agent, but he does not appear, in his cases generally, to have employed it extensively or vigorously.

The latter half of M. Bouillaud's first volume, and the whole of the second, are devoted to the description of the individual diseases which constitute the object of the work, each of which is illustrated by a copious collection of carefully-given cases; and each set of cases is preceded by some preliminary observations, and followed by a condensed history of the particular disease under consideration, an account of the actual state of our knowledge in regard to it, and the results of the clinical observations and experience of the writer himself. The cases of recovery, as being in some degree incomplete in an historical point of view, inasmuch as they are deficient in the pathological anatomy of the disease, are kept apart, and reserved for the termination of each article. Cases of this kind are, as M. Bouillaud remarks, too much overlooked by most modern French writers.

*Pericarditis.* Both Corvisart and Laennec, though so peculiarly distinguished by their tact and sagacity in the recognition of diseases, speak emphatically of the doubt and difficulty usually experienced in their attempts at diagnosticating this disease. The latter concludes his remarks on the subject by observing that pericarditis is a disorder, the existence of which, during the life of the patient, the most able physicians rather guess at than recognize. It is to the valuable memoir of M. Louis, which first appeared about twelve years ago, that we are chiefly indebted for the great advances which have recently been made towards the establishment of a correct and adequate diagnosis of this affection; and M. Bouillaud conceives that he has been able to contribute somewhat to the completion of this desirable object. Collin, Latham, and Stokes have likewise done good service in the same cause.

Our author has thrown his cases into groups, which are founded on the particular period of the disease existing at the moment of death, or rather on the precise character of the anatomical lesions discovered on dissection; a division which he thinks of more practical utility than the usual one, into acute and chronic.

His first group contains the cases of individuals who died in the period of inflammatory congestion, or in that of effusion or suppuration; the second comprises those in which the absorption of the coagulable lymph had commenced; whilst the third and last comprehends those in which the morbid product had been completely organized, and undergone a transformation into fibro-cartilaginous or osseous tissue. We must content ourselves with noticing the more general observations, although many of the individual cases are extremely interesting and instructive.

Amongst the chronic consequences of pericarditis, the most constant, judging from the cases, consist in morbid alterations of the muscular structure and dimensions of the cavities of the heart, (hypertrophy, dilatation, &c.,) and, above all, a diseased condition of the internal lining membrane and of the valves. Cases affording additional evidence to the same effect abound in the works of Corvisart, Tommasini, Hope, &c. There are still, however, writers who affirm that, in certain cases of simple chronic pericarditis, unaccompanied by any lesion of the valves or orifices, symptoms quite similar to those produced by a mechanical obstacle to the circulation occur. Our author has never met with such a one, and doubts altogether the correctness of observation on the part of those who advocate its possibility. He does not deny that a simple chronic pericarditis, even in the absence of any notable quantity of effused fluid, may offer some slight obstacle to the circulation and respiration, but asserts that any symptoms of this kind so arising are so much milder in degree than those depending on an internal obstruction to the course of the blood, as not to be capable of being confounded with them by any attentive and judicious observer. When the latter state of things, on the contrary, exists, it may, he thinks, be diagnosticated with as much certainty as a stricture of the urethra, or any other simple surgical disease; whereas, the former is much more obscure, and often at first only recognizable from the previous history of the case.

The anatomical appearances found on dissection are detailed at considerable length. The thickening often ascribed to the pericardium in these cases is, for the most part, rather apparent than real, depending on the effusion of one or more layers of coagulable and partially organized lymph over its surface, or on the hypertrophy of the cellular membrane beneath it. The pressure of the effused fluid, if considerable in quantity, or that of accumulated false membranes, sometimes leads to an atrophic state of the heart, —an effect analogous to that produced on the lung by the long-continued pressure of a pleuritic effusion.

Concurrently with the morbid condition of the pericardium, the internal lining of the heart is in many cases red, and somewhat thickened, and that most conspicuously in the *valves*, which are often swollen and infiltrated, or else present fungoid excrescences on their free edge; and coagula of blood, of a date evidently anterior to death, are often found in greater or less quantity within the heart. The muscular tissue of the heart likewise is liable, as well as the serous, fibrous, and cellular tissues entering into its structure, to become hypertrophied in some instances, and soft and friable in others. The softened muscle may assume either a deep red or brownish colour, or else a whitish or pale yellow. It is the latter of these two varieties which has alone been specified by Laennec, and compared by him to the effects which would be produced by maceration, to which it seems in fact to be in some degree owing; and M. Bouillaud remarks, that it is chiefly in those instances of chronic pericarditis where the effusion is almost entirely of a serous nature that it is observed. M. Désclaux, in several instances where he had produced inflammation of the pericardium by artificial means, on dissection of the animals so treated found the internal membrane of the heart likewise red, and the valves (and more especially those of the left side) thickened, swollen, infiltrated, and as it were fungoid on their edges.

The diagnosis of pericarditis may now, according to M. Bouillaud, be formed with certainty in a great majority of cases. The following is a condensed account of the symptoms, physiological and physical, enumerated by him.

1. Pain, more or less acute, below the nipple, or towards the lower end of the sternum, occupying occasionally the whole præcordial region, and sometimes darting towards the axilla and left arm, and at other times towards the diaphragm, epigastric or hypochondriac regions, and especially that of the left side. This pain, which is sometimes very acute and pungent, like that of pleurisy, is increased by percussion, respiration, and cough: it is sometimes so severe as to render it impossible for the patient to straighten the side, or even to lie on it. In many cases the pain is so obscure as almost to be overlooked, and is only complained of when pressure is made from below upwards in the epigastric region; and in some it is altogether absent. The coexistence of a very acute pleurisy, or of a violent articular rheumatism, has occasionally been sufficient to mask it entirely. Yet it is perhaps the *simplest* form of pericarditis which is the most commonly unaccompanied by pain, and which is hence the most frequently latent, as had been already observed by Laennec; who is here, however, opposed to Corvisart. When the disease originates in rheumatism, pain is sometimes not at all conspicuous, unless a pleurisy coexists, and then it is most violent when the pleuritic inflammation occupies the left side of the chest, and particularly the upper surface of the diaphragm.

2. The beat of the heart is generally increased in strength, and may be either regular or irregular, intermittent or tumultuous. Sometimes, however, the stroke of the heart is almost or altogether imperceptible, which seems to depend on the presence of a very profuse effusion of fluid in the pericardium, by which the sounds likewise are rendered distant and obscure. In one case of considerable effusion it was observed, that the place of the beat of the heart could be changed considerably by varying the patient's posture. When the period of organization is taking the place of that of inflammation, M. Bouillaud has sometimes observed a very singular phenomenon, not hitherto, so far as he knows, noticed by any one else; namely, the second motion of the heart is double, and accompanied by a peculiar, dry, hard sound, (*craquement.*)

3. The prominence of the præcordial region, pointed out by M. Louis, is a valuable sign, which our author has often had occasion to verify.

4. There is an increased extent of dulness on percussion in the præcordial region, which depends partly on the quantity of fluid effused, and partly on the inflammatory turgescence of the heart itself; but the former element has far the greater share in the production of this effect. This is a sign, however, which, like several of the others, can only be expected to be met with in a particular period of the complaint, as it diminishes with the progress of absorption: and in those cases where scarcely anything but a little coagulable lymph is effused, this symptom will hardly, if at all, exist, or only in relation to the turgescence of the heart. In fine, it is only when there is a considerable effusion of fluid that percussion affords us much assistance in the diagnosis of pericarditis; and here, by altering the patient's posture, the level of the dull sound can sometimes be varied at pleasure. When the quantity of fluid is small, no dulness may be perceivable till we place the individual either erect or sitting.

5. Auscultation, till lately, has done very little for this disease. The sign pointed out by M. Collin, and compared by him to the creaking of new leather, is not admitted by Laennec in his second edition. Its very great rareness may account for this: until recently, as already mentioned, neither Andral nor Louis had ever met with it. Within the last five or six months immediately preceding the publication of the present work, M. Bouillaud has encountered it two or three times; so that from this and from Mr. Mayne's recent paper, in which the evanescent nature of the symptom is dwelt on, we are inclined to think that in many cases it has not been discovered, because not sought for with sufficient assiduity within the short period of its natural duration. It seems to our author to require for its production a peculiar degree of density in the false membranes which rub against each other, and he thinks it may sometimes also depend on the dragging of the adhesions. The sound of friction is very common, and occasionally imitates the *bruit de scie,* or *bruit de râpe;* and M. Bouillaud has himself met with the true bellows murmur in at least six or eight cases.

He does not assent to that part of Dr. Hope's explanation of the bellows murmur, which ascribes it to the increased force of the heart's action; for it is absent in many cases of very violent palpitations, and sometimes present when the heart's motions are comparatively feeble. He fully concurs with him, however, in thinking that it may occasionally be produced by swelling of the valves; being convinced that it depends, for the most part, on the coexistence of endocarditis, and in some instances on the fibrinous concretions within the heart, which are now known not unfrequently to accompany this lesion. The compression of the heart by a large quantity of effused fluid is, he thinks, likewise sufficient to give rise to it.

It is to the general or physiological symptoms of pericarditis that what has been said by authors of the uncertainty of its signs chiefly applies; their variability depending on the degree of intensity of the disease, the number and severity of its complications, &c. Thus the pulse, which is generally much accelerated, may be either large and accompanied with moisture of the skin; or small, hard, and irregular, the skin being remarkably dry. There is occasionally dyspnœa, and an insupportable sense of oppression and anxiety, jactitation, and sleeplessness, and in some instances delirium and convulsions. The face, which may have been pale and sunken at the commencement, if the patient survives a few days, becomes often of a livid or violet hue, and the limbs swell. The suffering induced is often exquisite. Mirabeau, who, as above stated, fell a sacrifice to it in its most violent and acute form, is said to have entreated his attendants to shorten his life and his tortures by opium. The cases in which there is the most vehement reaction are those which are complicated with inflammation of the pleuræ, especially of that portion of the membrane which lines the diaphragm. The accompanying dyspnœa, when it exists in a very aggravated degree along with faintness and syncope, seems frequently to depend on a large effusion of fluid into the pleuræ or pericardium, and occasionally also on polypous concretions in the cavities of the heart. Frequent vomiting, from sympathy of the stomach with the inflamed organ, is amongst the occasional symptoms of pericarditis; and, in some of the complicated cases just alluded to, M. Bouillaud is disposed to attribute its occurrence to the proximity of the inflamed pleura covering the diaphragm to the peritoneum, and to the reaction of the diaphragmatic nerves on those of the stomach.

None of the symptoms above alluded to, taken *isolatedly*, can be considered conclusive as to the existence of pericarditis; but, whenever an individual is suddenly seized with feverishness, oppression, and anxiety, and there is smallness, irregularity, and intermittence of pulse, together with pain in the præcordial region, extensive dulness on percussion there, and the sound of friction or creaking within the pericardium, we may affirm with confidence the presence of this disease.

*Chronic Pericarditis* is generally only the sequela of the acute form. Sometimes, however, pericarditis bears a chronic stamp from the very first, being unaccompanied by any notable degree of fever, and in many of such instances it is latent; though, if a sufficient degree of care be employed in examining them, they may generally be detected. Thus, if, in addition to the physical signs indicative of effusion, there be some degree of pain or uneasiness in the præcordial region,—a slow fever, with or without exacerbations in the evening and after taking food,—a general sense of oppression,—slight œdema of the face, which is occasionally of a violet tinge, and swelling of the ankles increased by the erect posture, we may conclude the existence of a chronic pericarditis. The existence of adhesions of the pericardium to the heart is not rendered manifest by any diagnostic symptoms, as far as is known to our author. He has not hitherto succeeded in observing that species of undulatory motion which Sanders has asserted is perceptible to the hand applied to the epigastrium in such cases, and which is supposed by him to depend on the alternate drawing up and depression of the diaphragm, in consequence of the motions of the apex of the heart, now closely adhering to the pericardium. He does not at all agree with Corvisart in supposing that these extensive adhesions *must* derange the actions of the heart and diaphragm, and thus lead sooner or later to inevitable death. So frequent is the connexion of this disease with rheumatism, that he believes, as we have already seen, that, of any given number of acute rheumatic cases, at least one half present symptoms of pericarditis or of endocarditis, or of both united, at some period of their progress. The time of life most subject to it is that between ten and thirty. We agree with M. Bouillaud in thinking that Louis has certainly underrated the influence of cold as an auxiliary cause of this disease.

*Prognosis.* The prognosis in pericarditis generally is considered by our author in a much more favorable light than by almost any previous writer on the subject; and in this he appears to be justified by the frequent traces of it observed on dissection in individuals who have been carried off, long subsequent to its cessation, by other diseases; and he asserts that he has himself succeeded in subduing the majority of cases of it which have fallen under his care during the last few years; and amongst these successful cases there were even several where it existed in complication with pleurisy or pleuropneumonia. The average duration of the disease, when judiciously and successfully treated, may be estimated at from seven to fourteen days. Very violent cases of it may, however, prove fatal in little more than twenty-four hours. Those originating in rheumatism are generally the most obstinate.

*Treatment.* His treatment consists chiefly in free and frequent bloodletting, both general and local. Thus, for example, in the case of an adult in the vigour of life, he orders, on an average, from three to four venesections, to the amount of twelve or sixteen ounces each, within the three or four first days; together with the simultaneous application of twenty or thirty leeches, or cupping to the extent of eight or twelve ounces during the same period. In very feeble patients, where bloodletting seems inexplicable, or has already been carried as far as is safe, we observe that he sometimes employs diuretics or purgatives, but only in that cautious and distrustful manner which characterizes the modern French school. When, notwithstanding all the measures put in practice, the disease unfortunately passes into the chronic stage, local bleedings are still occasionally employed, in combination with counter-irritants, —blisters, cautery, moxa, setons, or frictions with mercurial ointment, tartar emetic, or croton oil. A very low diet is at the same time observed, and tepid baths are employed. When all these fail to produce the absorption of the effused fluid, it has been proposed to attempt the relief of the patient by means of a surgical operation. M. Bouillaud discusses the operation more at large when treating of hydropericarditis. When the motions of the heart are irregular and tumultuous, and hypertrophy of this organ has supervened, digitalis deserves a trial; but, when atrophy of the heart has been induced by the presence of the effused fluid, the employment of the same remedy might be attended with dangerous consequences.

We cannot quit the subject of treatment without once more protesting against his almost total neglect of those potent auxiliaries, especially in the early stages of the disease, calomel and opium, as there are few inflammations over which their influence has been more satisfactorily made out than in that under consideration; nor are we inclined to think that he does justice to the virtues of the solution of tartrate of antimony, as employed by Laennec and others in serous inflammations, and which, in some of the cases even in the work before us, (the 31st and 32d, for example,) seems to have been productive of signal benefit; whilst, in cautious hands, it very rarely induces that dangerous irritation of the mucous membrane of which he is so apprehensive: and, when we take fairly into consideration the comparative degrees of danger attendant on inflammation of a serous membrane and that of a mucous one,—the importance of the organ enveloped in the former,—the difficulty of getting rid of the effusion by which nature here endeavours, though imperfectly, to relieve herself,—and the dangerous nature of the sequelæ, we become reconciled to incurring the risk which must always be more or less connected with the employment of so powerful an agent. It is, however, chiefly in the very earliest stage that we are disposed to place much reliance on it.

Of the results of his treatment our author gives the following summary. Of eighteen cases of acute pericarditis, twelve were cured, and six ended fatally; but, of these last, three were not treated on his recent system of very free bloodletting, and, of the remaining three, one died of tetanus, quite independently of the pericardial inflammation; so that there were only two deaths to set off against twelve recoveries; and, of these two, one was complicated with a most violent pleurisy, together with pneumonia, splenitis, and erysipelas of the face; and, on dissection, the pericarditis was found to have almost entirely disappeared. In the other case, complications likewise existed. From all this he thinks himself justified in concluding that simple pericarditis would hardly ever be fatal, if early and properly treated.

*Endocarditis.* In the chapter devoted to endocarditis, or inflammation of the membrane lining the cavities of the heart, which M. Bouillaud looks upon as the most original and important portion of his work, he sets out by assuring us that the opinions here put forth in relation to this affection are the result not of any preconceived theories, but of a fair generalization of facts. Its very existence seems to have been unknown to Corvisart and most other writers on the subject of diseases of the heart. Baillie, however, speaks of thickening of the valves, accompanied by the loss of transparency and the assumption of an opaque white colour; of the lining of the ventricles being frequently a good deal thickened, and appearing like a firm white membrane; and adds, "I have also seen the valvular apparatus between the auricle and ventricle in a state of inflammation, and covered with a layer of coagulable lymph; but this I believe to be very uncommon." Burns, too, had found the internal surface of the right auricle covered with a layer of flocculent lymph; and that of the left, in another case, partly ossified, and lined by a false membrane. Kreysig attributed the frequent formation of polypus concretions within the heart to the existence of inflammation within this organ; an opinion which was subsequently impugned by Laennec, who asserts that inflammation of the internal membrane of the heart and great vessels is infinitely rare. As for the redness occasionally found in them, he looked upon it in a vast majority of cases as a mere cadaveric phenomenon; and especially so where the agony preceding the fatal termination has been greatly prolonged, and accompanied by a state approaching to suffocation, or where there is an evident alteration in the constitution of the blood, or signs of incipient decomposition in other parts of the body. At the very most, he would admit the probability of such inflammation in those cases only where the redness is accompanied by swelling, thickening, or infiltration; whilst he considers the existence of ulceration, or of layers of coagulable lymph, as the only unequivocal proof of its reality. Liquid pus can, for obvious reasons, rarely be expected to be met with in such a situation. Little has been added to our knowledge on this point by Dr. Hope's work; the opinions advocated in it coinciding very nearly with those of Laennec just detailed.* The extensive investigations made by M. Bouillaud during the last three years enable him to state with confidence, even in opposition to the high authority just cited, that inflammation of the lining membrane of the heart is a very common affection, and at least as frequent as pericarditis itself. Its early stage, he admits, is not attended with such incontestable anatomical evidences as is that of the last-mentioned diseases; but those of the more advanced periods are altogether analogous, and quite as satisfactory. When redness of the membrane in question is detected, he does not by any means conclude from that circumstance alone that there must have been inflammatory action, but is much guided in the formation of his opinion by the symptoms which characterized the disease during life. Of the possibility of such an affection we have positive and altogether indubitable proof in those cases where inflammation of the veins from external injury has gradually extended to the lining membrane of the heart, as well as an instructive and unimpeachable example of the appearances properly characteristic of such inflammation.

In case 43 we have an instance of this disease existing in conjunction with pericarditis and hypertrophy of the heart, with thickening of the valves of the left side. The patient was a young man, of twenty years of age. The leading symptoms were dulness

* It will be seen in the notice by us of another work by M. Bouillaud, ("Sur le Rheumatisme,") that the frequency of this affection of the lining membrane of the heart had been recognized and publicly noticed by Dr. Watson, of London, previously to the publication of M. Bouillaud's observations on the subject.—Eds.

on percussion and prominence in the præcordial region, together with a vibratory sensation imparted to the hand applied over it. A sound of friction diffused over the cardiac region, and synchronous with the motions of the heart, was also heard; whilst there existed, moreover, a distinct bellows murmur during the ventricular contraction, and this manifested its greatest intensity over the left auriculo-ventricular origin. The pulse was eighty, small, and intermittent; and the face and extremities were œdematous. The patient was easily put out of breath, and complained of a slight ill-defined pain in the chest. The diagnosis formed from this group of symptoms by M. Bouillaud was "acute endocarditis, grafted on an old lesion of the valves of the left side, and general hypertrophy of the heart;" and the appearance after death (which occurred on the fifth day from the supposed commencement of the inflammation of the inner membrane,) fully justified his prediction. About four or five days before the fatal termination, considerable cerebral oppression supervened, with temporary loss of consciousness, convulsions, and tremendous palpitations, the heart striking like a hammer against the side of the chest. On dissection, in addition to hypertrophy and the false membranes of recent pericarditis, the tricuspid valve was found slightly thickened, and a colourless coagulum (which he supposes to have been formed previous to death, namely, at that period when the cerebral symptoms supervened,) occupied both ventricles. The mitral valve was very much thickened, reddish, and of a fungoid appearance, and presented inequalities on its surface, which were obviously of an old date. The columnæ carneæ were greatly thickened, and one division of the valve adhered to the wall of the ventricle. The internal surface of the left auricle was lined with a false membrane, which adhered to it pretty firmly. Two or three spoonfuls of a reddish fluid were found at the base of the brain, and the arachnoid was much injected, and obviously thickened over the left anterior lobe; and the substance of the brain, on section, presented numerous, thickly strewed, bloody spots. The case just detailed being unfortunately a complicated one, as are indeed all those in this section, is far from affording us satisfactory information as to the peculiar symptoms which are to be considered characteristic of endocarditis. In the 50th case, in addition to the redness of the internal membrane of the heart, there existed numerous small ulcerations over the commencement of the aorta and its valves: the symptoms are but very inadequately recorded, and the case was as far from being a simple one as the last, inasmuch as an extensive pleuropneumonia coexisted.

His second division of cases of endocarditis embraces those in a more advanced period, and comprises eleven cases, of which the first four present us with examples of adhesion of the valves to the internal surface of the heart; a species of lesion which, though so important in its effects, (hypertrophy, dilatation, &c.) has hitherto been almost entirely overlooked. The next three are instances of organized false membranes of various extent on the inner surface of the organ, which resemble in appearance the white patches so often found in the pericardium, and are, like them, of an inflammatory origin. The last four cases are examples of granulations or vegetations on the valves; a species of lesion which becomes of importance as soon as it exists to such a degree as to impede their action or materially to obstruct the orifices.

The third division of cases of endocarditis comprises those in the most advanced stage, the first subsection consisting of those where there exists induration and thickening of the valves, without any notable contraction of the orifices. Where this morbid alteration in the valves is carried to a great extent, it unfits them for the adequate fulfilment of their office, and thus, by permitting regurgitation, paves the way for hypertrophy, dilatation, and other diseased conditions of the organ. In the second subsection are contained cases in which a similar state of the valves exists, but accompanied by considerable narrowing of the orifices. In one of these cases the hydriodate of potass was administered in half-grain doses daily, with a view to combating the symptoms of hypertrophy of the heart which were present; but, as it seemed only to raise the pulse and to exasperate the patient's condition, it was soon abandoned.

Of forty-seven cases of fibro-cartilaginous and osseous induration of the valves, one was in an infant of only ten months old; two were in children of seven and ten years of age; twelve in individuals of various ages, from sixteen to thirty years; fifteen were between thirty and fifty; eleven between fifty and seventy-two; and in three the ages are not mentioned. Thus, out of forty-four cases where the age was ascertained, no less than thirty-three were under fifty years of age; a fact which is certainly rather favorable than otherwise to the theory which insists on the very frequent inflammatory origin of these lesions.

In his general remarks on endocarditis in its first or inflammatory period, which is characterized by sanguineous congestion, softening, ulceration, and suppuration, he states that redness of the membrane lining the cavities of the heart was met with in twelve out of thirteen cases; and he attributes its absence in the thirteenth to the short time the disease had existed, its disappearance under these circumstances being quite analogous to what takes place in regard to slight erysipelatous redness immediately after death. The colour, when present, varies from a slight rose tint to a scarlet, violet, or even a brownish hue, is partial or general, and is frequently confined to the valves alone; and, even if universal, is most intense on them. He believes it to depend rather on a tinging of the membrane than on true capillary injection; it does not usually penetrate beneath the membrane. It cannot readily be washed away, but yields to prolonged maceration; and M.

Bouillaud has taken occasion to express his dissent from the opinion of M. Cassimir Broussais, who thinks that we may distinguish the tinging of the lining membrane of the sanguiferous vessels which takes place after death from true inflammatory redness, by the effects of maceration. The frequency of cadaveric imbibition, as it is called by Laennec, is fully recognized by our author, especially after typhoid and putrid diseases, as well as when the examination of the body has been postponed till decomposition has commenced. Yet, on the other hand, notwithstanding the high authorities which may be cited against him, he is satisfied of the inflammatory nature of that redness which is discovered, prior to the appearance of any trace of decomposition, in an individual who during life has presented the train of symptoms which he believes to be characteristic of endocarditis; and the fact becomes altogether incontrovertible when the redness coincides with swelling, thickening, and infiltration of the parts which it occupies, or when purulent matter, or a pseudo-membranous effusion, or even colourless and adhering coagula, resembling in nature the buffy coat of the blood, are detected; and especially when this tinting coincides with a similar redness in the blood-vessels, they having been ascertained to be in a state of inflammation by the symptoms during life. A redness similar to that in question was produced, be it remembered, in this same membrane, in those experiments on animals where inflammation of the pericardium had been artificially induced by mechanical or chemical stimulants. The thickening of the membrane is rarely very obvious, save on the valves. Its inflammation imparts to the blood contained within the cavities of the heart a tendency to coagulate; a phenomenon which is also exhibited in the blood contained within an inflamed artery or vein. The fibrinous concretions consecutive to an endocarditis are white, colourless, elastic, and glutinous, adhering to the walls of the heart, and twisted round the tendons of the valves and fleshy pillars. They are, according to our author, in a state of imperfect or incipient organization; and he conceives it possible that small adhering fragments may even become perfectly organized, and so give rise to vegetations or granulations on the valves. The coagula resulting from mere obstruction to the circulation, on the other hand, are soft, reddish, or of a darkish hue, and bear a close resemblance, both as to consistence and colour, to currant jelly.

As to the morbid appearances in the second period of the disease, he remarks that the vegetations or granulations display a peculiar preference for the valves, and especially for their free edges; and adopts M. Laennec's division of them into the globular and the warty. The former, which are soft, easily detached, and of a whitish, yellowish, or reddish hue, would have been better designated by the name of albuminous or fibrinous vegetations. He believes they sometimes originate in the organization of effused and adherent coagulable lymph; being thus analogous to the granulations met with on the surface of the pericardium, pleuræ, or peritoneum, when in a state of chronic inflammation. The warty excrescences, on the other hand, are of a horny or cartilaginous consistence, and very firmly attached. These vegetations vary in size from that of a grain of millet to that of a small pea, and may exist either separately or congested into groups, so as to assume a cauliflower appearance. They rarely exist as an insulated lesion, being very commonly accompanied by fibro-cartilaginous or calcareous induration of the valves, and, when very large and confluent, necessarily themselves give rise to considerable contraction of the orifices and impediment to the valvular actions.

Of the cartilaginous and osseous degenerations which characterize the third period of the disease, the most frequent seats are the fibrous zones surrounding the orifices of the heart, together with the adjacent valves. The ossifications occasionally assume a very irregular shape, and sometimes penetrate deep into the muscular substance of the heart itself. The diseased valves are in some instances perforated with holes, torn, and reticulated; and, in one of the cases here detailed, one of the aortic valves was found almost entirely detached and floating in the cavity of the vessel; a species of lesion which is by no means unprecedented. The contraction of the orifices is perhaps the most serious of all the morbid alterations consequent upon endocarditis. In its extreme degree, the point of the little finger, or even a quill, can scarcely pass. When the thickened and indurated valves become consolidated with each other, a permanent opening is formed, of a roundish, oval, or slit-like outline; which, in the case of the auriculo-ventricular valve, from its projection into the body of the ventricle, is compared to the glottis, or, when much thickened, to the os tincæ. This narrowing of the passages of the heart by chronic inflammation is very analogous to what takes place in other organs of the body under the influence of the same cause,—as in the urethra, and lachrymal and biliary ducts, the œsophagus, the cardia, the pylorus, the rectum, &c.; and the hypertrophy of the heart which succeeds may be compared to the thickening of the muscular coats of the bladder, stomach, and other hollow organs, which arises in such circumstances, from the difficulty of expelling their contents in consequence of the obstruction. The inflammatory origin of the cartilaginous and osseous depositions within the heart, in a great number of cases, is now fully recognized by Andral and many of the best pathologists of the day. All the morbid alterations under consideration are much more common in the left side of the heart than in the right, though they are by no means confined to the former, as Bichat insinuates.

Inflammation of the endocardium is rarely indicated by any peculiar pain; and, when this symptom does exist, it is generally referrible to the coincidence of pericarditis or pleurisy, rather than to the affection in question. There is, however, often a considerable sense of uneasiness, anxiety, and oppression, referred to the præcordial region; and this, in extreme cases, is accompanied by a tendency to syncope, the pulse generally becoming very rapid; and it is then for the most part irregular and intermittent. Palpitations are commonly present. The impulse of the heart against the side of the chest is usually very strong, and is felt over a greater extent than natural; which M. Bouillaud ascribes to the turgescence of the organ, under the stimulus of the internal inflammation. The extent of præcordial dulness on percussion is found to be somewhat increased: the dulness arising from this cause may, however, be distinguished from that depending on a simple effusion into the pericardium, by observing that, in the case of endocarditis, the beat appears superficial, being visible and perceptible to the hand; whilst, if there be considerable effusion, on the other hand, it is deep-seated, and scarcely at all to be recognized by the sight or touch, at least when the patient is in the supine posture.

The *bruit de soufflet*, or bellows sound, which accompanies endocarditis, and masks one or both of the normal sounds, will vary in intensity, he conceives, according to the degree of swelling of the valves, and the abundance of the fibrinous or pseudo-membranous matter formed within the cavities of the heart. The pulse is often not in harmony with the heart's action,—the former being small and feeble when the latter is violent and tumultuous; a discordance which he ascribes to the presence of a considerable mass of fibrinous concretion in the heart, and to swelling of the valves and consequent obstruction of the orifices; and it is in such instances that we meet with extreme paleness of countenance, anxiety, and jactitation, swimming in the head, syncope, &c.; and, in the advanced stage of such cases, the venous circulation becomes impeded by the same circumstances, as is evinced by the bluish or livid colour of the face and extremities, together with an œdematous state of the same parts: and he is even inclined to think that the cerebral symptoms occasionally met with, the sudden loss of consciousness, the slight convulsive motions, the stertorous respiration, and foaming at the mouth, are the result of the congestion of the veins of the brain thus induced. When the obstacle to the circulation is considerable, but only then, great dyspnœa is present, attended with extreme restlessness and uneasiness in every position.

The above description of the symptoms is applicable to those cases only where the disease exists in a very acute and extensive form. When it is only partial, subacute, or chronic, its signs are much less marked, and to recognize it is, as he confesses, a matter of considerable difficulty, and requires great attention to the examination of the patient. The decidedly acute form can hardly be confounded with any other affection, except it be pericarditis; but our author admits that there are occasionally cases where he remains in doubt between these two diseases. Practically speaking, however, the difficulty is of little importance. When the pericarditis is accompanied with a copious effusion of fluid, the mistake, he thinks, could hardly be committed, if we attend to the diagnostic symptoms mentioned above. Where, however, such effusion is not present, the difficulty becomes seriously increased. Our own impression is, that, in the present state of our knowledge, the distinction will often be impossible. The friction between the opposing layers of the pericardium, when roughened by the presence of adherent coagulable lymph, produces a morbid sound much resembling that heard when the valves are inflamed and thickened. If, however, this sound cease on change of posture, as is sometimes the case, our author is then inclined to refer it to the pericardium. The existence of "frémissement cataire," with intermittence or irregularity of the heart, are also signs of great value in diagnosticating a narrowing of the orifices. The triple or quadruple motion of the heart, already alluded to as one of the most remarkable interruptions of the natural rhythm of the organ, is dependent on this narrowing, or on induration of the valves; and the dulness of sound on percussion, which arises in the advanced period, depends on the dilatation and hypertrophy to which the lesions just mentioned eventually give rise. It is to auscultation chiefly that we must look for aid in our attempts to form the above diagnosis. The bellows murmur, the saw and rasp sound, are here signs of the first importance: out of above one hundred cases, some modification of these sounds was detected in all save one, and that one was not adequately examined. Induration of the valves is rarely accompanied with pain. In some instances a slight uneasiness or weight is felt at the præcordia or epigastrium; in nearly all, palpitations and a tendency to syncope are complained of. During the palpitations the pulse is of extreme frequency,—from 140 to 160 in a minute.

When, therefore, an individual presents to our observation a permanent "bruit de soufflet" in the præcordial region, a rasp-like or a sawing sound, whilst a vibratory sensation is imparted to the hand, and tumultuous action of the heart or palpitations and irregular pulse exist, it is in the highest degree probable, if the disease dates back some months or years, that we have to do with induration of the valves and narrowing of one or more of the orifices of the heart; and this becomes certain when to these local signs the general symptoms of impeded circulation are added; as, for instance, dilatation of the superficial veins; pulsation of the jugulars, synchronous with the pulse, from reflux from the right ventricle, in consequence of inadequacy of the tricuspid valve; violet tint of the complexion; congestions of the lungs, liver, mucous membrane, brain, &c.; passive hemorrhages or serous effusions; dyspnœa on slight exertions; cerebral derangements, loss of sleep, frightful dreams, &c.

M. Bouillaud next investigates the question as to whether it be possible to decide in which of the orifices of the heart the narrowing is seated; a point, the determination of which being, as he justly remarks, more curious than useful, we shall not at present dwell upon.

The symptoms arising from the adhesion of the valves to the parietes of the heart are much the same as those from contracted orifices; the reflux producing very similar local symptoms, and an embarrassment of the circulation to a nearly equal amount. A hardened and distorted condition of the valves is perhaps usually in such cases a preliminary stage, which gives rise to reflux of the blood; and this, in its turn, by throwing the valves back, facilitates their adhesion. He endeavours, indeed, to discriminate between the symptoms of these two morbid conditions, though, we think, not very successfully. Thus he believes that, in the case of adhesions, the *bruit de soufflet* is of a fuller character, and less grating or rasp-like; that the beat of the heart is less irregular, and the *frémissement cataire* less marked; that the pulse is not so small; and, lastly, that the dyspnœa, the venous and serous congestions, exist in a less degree.

The vegetations on the valves, when they do not interfere with their action or notably diminish the orifices, are attended by no very obvious symptoms; yet M. Bouillaud is inclined to suspect their existence when there remains a well-marked *bruit de soufflet*, unaccompanied by any other symptom.

Endocarditis is induced by the same kind of causes as pericarditis. It may occur either as a primitive or as a consecutive disease. The affections on which it is most apt to supervene are pericarditis, pleurisy, phlebitis, and acute rheumatism. In the acute stage, it may terminate fatally in a very few days; and one of the principal causes of death here is supposed to be the extensive formation of sanguineous concretions within the heart. If actively and properly treated, it will generally end favorably within about a week; but, if it pass into the chronic state, its duration is indefinite: yet even here the lesions thus produced become sometimes apparently stationary, and, under judicious management, life may be prolonged to a very advanced period.

On its treatment we shall not dwell, as it is essentially the same as that of pericarditis; or, if there be any difference, it is only that copious and repeated depletions are still more imperatively called for here, in order to prevent the coagulation of the blood, the deposition of false membranes within the heart, and permanent derangement of its mechanism. If it passes into the chronic stage, moderate local and general bloodletting, counter-irritation, diuretics and purgatives, digitalis, baths, absolute repose, and a very low scale of diet, are our chief resources. With regard to digitalis, we may remark that our author seems to have a peculiar preference for the endermic method of exhibiting it, denuding the skin by a blister, and then sprinkling from eight to twenty grains of the powder daily over the exposed surface. The syrup of asparagus is also amongst his favorite remedies.

*Carditis.* Carditis, or inflammation of the muscular and cellular tissues of the heart, is the subject of the next section. The existence of any well-authenticated and well-described instance of a general inflammation of the heart was doubted by Laennec. Partial inflammation, characterized by abscess or ulceration, is, he admits, of occasional occurrence. Ramollissement of the heart is placed by M. Bouillaud, as well as previously by Corvisart, amongst the occasional consequences of inflammation; though he is far from asserting, as Laennec insinuated he did, that ramollissement of this organ can have no other source. The induration of the muscular tissue of the heart has always been reckoned amongst the possible consequences of its inflammation. No authentic instance of gangrene of the heart is yet upon record. Carditis so rarely exists in an uncomplicated form, that Corvisart never met with an example of it. He has given, in addition to three cases of his own, six from Meckel, Storck, and Hildanus; and in all the latter pericarditis coexisted. M. Bouillaud has collected together several additional cases of a similar kind: amongst these is one by Dr. Latham, of a patient who died in two days of an acute inflammation of the heart. On dissection, the heart was found of a reddish brown colour, and much softened. On making an incision into the walls of the ventricles, there oozed out here and there between the muscular fibres innumerable drops of pus; a case which sufficiently proves both the extent to which inflammation may affect the heart, and that ramollissement is really one of its terminations. Mr. Stanley's case of true carditis was an equally decisive one, and must be in the recollection of most of our readers.

M. Bouillaud arranges his cases of carditis under the three following heads: 1st, those terminating in ramollissement or suppuration; 2d, those terminating in ulceration, perforation, and rupture of the walls of the heart, of the columnæ carneæ, tendons, or valves; 3d, those ending in ulceration, with the consequent formation of aneurism; 4th, those leading to induration in various degrees up to a fibro-cartilaginous or cartilaginous consistence, or even to perfect ossification; as in Burns' remarkable case.

A general carditis has never yet perhaps been met with in its simple state, but always complicated with inflammation either of the inner or outer membrane of the heart, or both. According to our author, ramollissement and suppuration of the heart are similar lesions, differing from each other only in degree. The colour of the softened structure is either reddish or yellowish white; the first indicating an earlier, the second a more advanced period of the affection, or that where purulent infiltration has taken place, analogous to the two successive conditions of the lung in pneumonia, or of the psoas muscle in psoitis. The purulent matter may be either encysted or infiltrated, and may make its escape either towards the external or the internal surface of the heart, though he is not aware of any recorded instance of the latter.

Ulceration of the heart, together with the aneurismal tumours and perforations which are consecutive to them, are much more frequent in the left than the right side of the organ. According to M. Breschet, the apex of the left ventricle is the most usual seat of these aneurismal cysts; but M. Reynaud has thrown some doubt on this generalization, as he, on the other hand, has found that, in seven out of thirteen cases analyzed by him, the disease did not occupy the apex. Perforation of the heart is much less apt to occur from rupture of these aneurismal tumours than by simple ulceration; the firm adhesion and thickening of the two layers of the pericardium forming, for the most part, a bulwark in the former case. When perforation does at length take place, it is generally rather by rupture than the absolute penetration of the ulcer. If the external wall of the heart be thus perforated, instant death, by bleeding into the pericardium, is the result; whereas, if the opening affects the septum, the mixture of the black and red blood is the only immediate consequence.

Corvisart was unable to fix on any diagnostic symptom by which to distinguish carditis from pericarditis; and neither Laennec nor our author has been more successful, which, inasmuch as the former is scarcely ever met with uncomplicated, is not surprising. The symptoms of aneurismal tumours of the heart, of which about eighteen cases are on record, are equally obscure, and the affection has not yet in any one instance been detected.* Yet M. Bouillaud considers that, with the joint aid of percussion, auscultation, and the touch, carefully and skilfully exercised, it is not altogether to be despaired of. Thus, it is evident that such lesions must produce a degree of dulness on percussion in proportion to their magnitude; that they may, in some extreme cases, eventually raise the corresponding portion of the thoracic parietes; and it is probable that an anormal sound may be produced by the entrance and expulsion of the blood through the opening of communication with the heart.

*Hydropericardium.* This disease is judiciously divided into active and passive; a distinction neglected by Corvisart. The latter being the mere result of an obstacle to absorption and to free venous circulation, in consequence of the primary disease of the heart, is not dwelt on. The former was considered by Laennec, as it is also by our author, as being extremely rare; and, if we

* In the case of the celebrated Talma, though he was attended by several of the most eminent medical men in Paris, the existence of this disease was never once suspected till revealed by the dissection. An aneurismal tumour, capable of containing a small hen-egg, was found at the apex of the heart: it communicated by an aperture, or cartilaginous ring, of about an inch in diameter, with the left ventricle; on the outside of this cyst the two pericardial layers were adherent, and formed the chief portion of its parietes. It was filled with fibrinous coagula, arranged in a succession of thin concentric layers. The immediate cause of his death was a neglected stricture of the rectum.

leave out of view those cases where the serous effusion accompanies or follows a purulent or pseudo-membraneous one, and where other evidences of pericarditis coexist, they have been unable to adduce any indubitable instances of it. The quantity of fluid which should be considered sufficient to constitute a dropsy of the pericardium seems quite unsettled: Corvisart fixes on six or seven ounces as the minimum. Laennec has laid down nothing very definite on the subject. Bouillaud thinks that Corvisart has placed the mark too high, as he conceives that the quantity of water which can be accumulated within this membrane, as the mere effect of a prolonged agony previous to death, rarely exceeds one or two ounces. When, however, hydropericardium is well marked, the quantity is often so considerable as to leave no difficulty, as it amounts occasionally to two or three pounds; and, in one case of Corvisart's, it amounted to no less than eight pounds. In opposition to Laennec, our author asserts that the heart has occasionally, in such cases, a whitish macerated appearance, from the long-continued contact of the fluid. He differs also from the same distinguished writer in respect to the difficulty of recognizing its existence during life, and believes that it may be detected by its physical signs in nearly every case where the effusion is somewhat considerable. The sounds of the heart in such instances become distant and obscure, and its beat, if felt at all, is (as Corvisart has remarked,) very variable as to the place where it is perceptible; being sometimes on the right and sometimes on the left side of the chest, and occasionally disappearing altogether when the patient is lying in a supine posture. As for the fluctuation seen by Senac, and felt by Corvisart, M. Bouillaud's own experience rather inclines him to doubt the propriety of placing it amongst the symptoms of this disease. In the only instance where he felt anything like fluctuation, he had reason to think it depended on the application of the heart itself, in its contraction, to the parietes of the chest over an unusually large surface, in consequence of its displacement by a tumour within the left side of the thorax. The prominence of the præcordial region is insisted on, and with reason, by Corvisart, who first noticed it. Its treatment, when it is discovered, will be the same as that of other active dropsies,—bleeding, diuretics, purgatives, &c. With regard to the propriety of paracentesis thoracis in any case of this disease, the data hitherto known to M. B. are insufficient to enable him to decide; and, as to the still bolder proposition of Richerand, to inject the pericardium with a stimulant fluid, in order to produce, as in the parallel practice in hydrocele, an artificial inflammation of the serous membrane, adhesion, and a permanent cure, he is still less in a condition to be justified in countenancing it.

*Hypertrophy of the Heart.* This affection, which was considered by Corvisart in a point of view subservient to its dilatation, as is evident from his nomenclature of heart-diseases, has been placed in a somewhat original and truer light by Bertin, who divides its varieties into three kinds,—the simple, the eccentric, and the concentric. The last of these seems to have been altogether unnoticed previously, though in reality it is less rare than the simple variety.

According to M. Bouillaud, hypertrophy of the heart hardly, if ever, exists in an insulated form; being almost always complicated with chronic pericarditis or endocarditis, or their consequences; and, where the valves and orifices of the heart are in a healthy condition, it does not give rise to any such train of symptoms as has been ordinarily but erroneously ascribed to what was formerly called active aneurism of the heart. The connexion of cerebral apoplexy with hypertrophy of the left ventricle, first perhaps pointed out by Legallois, and powerfully advocated by Richerand and Bricheteau, is well made out by numerous cases in the present work. Of fifty-four cases of hypertrophy here detailed, eleven (that is, above one-fifth,) present the coincidence of cerebral hemorrhage or of softening of the brain; and this in individuals of various ages, from twenty-five up to seventy-nine. M. Bouillaud falls into an error when he supposes that he is the first to have noticed the frequent coexistence of ossification of the cerebral arteries with apoplexy, as it is mentioned very expressly, and at some length, by Dr. Baillie, who views these phænomena in the relation of cause and effect. The opinion was subsequently alluded to, and we think successfully combated, by the late Dr. Cheyne, in his valuable treatise on Apoplexy.

Destruction of the eye by inflammation has been placed by Testa and Corvisart amongst the occasional consequences of organic diseases of the heart; but there is but a single case as yet, as far as we know of, adduced in support of the opinion. Gangrene of the limbs has also been ascribed to the same cause; but, in all the cases which have fallen under M. Bouillaud's notice, the gangrene has appeared to originate, not as a direct consequence of the heart-disease, but from obstruction of the artery leading to the part by means of a coagulum: whether, however, this state of the blood be the cause or the consequence of the gangrene, admits, we think, of question. In the 131st case, both the vein and the artery were thus obstructed. Though hypertrophy of the right ventricle is acknowledged to be very much rarer than that of the left, Laennec was evidently in error when he states that it never reaches a high pitch, as M. Bouillaud has mentioned three cases in which the parietes of the right ventricle varied from eight to sixteen lines in thickness. The concentric hypertrophy, or that with diminution of the cavity affected, may be explained by supposing it to originate in inflammation of the inner membrane of the heart, which will probably affect most especially the more internal muscular layers, and thus lead to their disproportionate development, and consequent encroachment on the interior of the ventricle. When, on the other hand, there is great dilatation, together with hypertrophy, it is usually dependent on obstacle to the circulation from a diseased state of the orifices, and is generally first apparent in the cavity just behind such obstruction. So frequent is the coincidence of hypertrophy with chronic pericarditis or endocarditis, and their consequences, that, when the latter exist, we may almost unhesitatingly conclude that the former is present also. Thus, out of thirty-three cases of these membranous inflammations given in the present work, which terminated by induration, thickening, &c., hypertrophy was not absent in a single one. To the circumstance of the thickening of the muscular structure of the heart being so much more conspicuous than that of the membranous, in their respective chronic states, may be ascribed the almost exclusive degree of notice which the former has met with: and yet there are many analogous instances where the inflammation of the lining membrane of other hollow muscular apparatuses (as the stomach, intestines, bladder, &c.) eventually gives rise, by means of long-continued irritation, to hypertrophy of the adjacent muscular fibres; and the analogy might be pursued still further, by observing that, in these cases likewise, an obstruction to the free course of the matters contained within them often exists, and forms a link in the chain of cause and effects, giving rise in some cases to dilatation, and in others to contraction.

The idiopathic signs of hypertrophy of the heart consist in the permanent augmentation of the force and extent of its pulsations, and a consequent augmentation, according to M. Bouillaud, of the intensity of the double sound. If to these we add an increase in the extent to which dulness is perceptible on percussion in the præcordial region, and occasionally a notable prominence in the same part, he thinks we have enumerated all its proper signs. The whole mass of the heart, instead of the mere point, seems to come into contact with the side of the chest at each beat. The pulsations in hypertrophy, it is further stated, are irregular, save in such cases where contraction of the orifices or a nervous affection of the heart coexists with it. Muscular substance being a bad conductor of sound, when the thickness of the walls of the heart is excessive, (twelve to fifteen lines and upwards,) and the cavities are at the same time diminished, the double sound is, he confesses, rendered somewhat obscure, and as it were smothered; but, where this thickening is moderate, and the capacity of the cavities is not lessened, or is, on the contrary, increased, these sounds become stronger and clearer, and are audible over a greater extent of the chest than natural; the sound produced by the impulse of the heart against the side having somewhat of a metallic ringing character. The bellows-sound, it is asserted, is very rarely present in cases of simple hypertrophy, and then only during the existence of palpitations. So very rare is it in such cases, that its existence should, in almost every instance, lead us to suspect some complication of disease about the valves or orifices. The extent to which dulness on percussion is heard is in the joint ratio of the hypertrophy and the dilatation. The prominence of the præcordial region, which is sometimes obvious to the eye and touch, and susceptible of accurate measurement, was, we believe, first noticed by our author, who gives several cases where it existed, and in which there was evident increase of space between the ribs of the left side. The pulse in the simple and in the aneurismal variety of hypertrophy of the left ventricle, in their uncomplicated state, is strong, large, vibrating, and regular. In the concentric variety, or that with diminution of the cavity, the pulse is vibratory, but small and cord-like. In all there is a tendency to active hemorrhages; the animal heat is slightly increased, the eye brilliant, and the colour florid, with occasional flushings. Where, however, complications exist, the above characters of the pulse are not to be expected; and it is in the latter cases only that passive serous and sanguineous effusions and congestions take place.

In uncomplicated hypertrophy of the heart, the respiration is not materially affected till the organ has acquired such a volume as to encroach on the lung; yet the great majority of writers on cardiac diseases have ascribed to hypertrophy, or active aneurism, such symptoms as the following; a violet hue of the face, and general congestion of the venous capillaries; passive dropsies and passive hemorrhages, dyspnœa, &c.; but these are in reality only so many signs of mechanical obstacle to the circulation, and indicate disease in the orifices, valves, &c.

In the present state of our knowledge, M. Bouillaud thinks it impossible to discriminate hypertrophy of the auricles during life; yet it so generally accompanies that of the ventricles, that he asserts we may, for the most part, conclude the existence of the former when we are able to recognize that of the latter. There seems to be a considerable probability that pulmonary apoplexy is occasionally dependent on hypertrophy of the right ventricle; but the three cases given in support of this view in the present work cannot be considered as at all conclusive in themselves, inasmuch as hypertrophy of the left ventricle coexisted in two of them.

In two cases detailed by our author, hypertrophy of the heart appears to have assumed an acute form, the period of obvious symptoms not exceeding three or four weeks; the patient in one instance being twenty-two, and in the other about forty years of age: but these cases, as it is very prudently admitted, want the additional confirmation of future ones, as they were not, as is obvious on their perusal, unexceptionable; and M. Bouillaud himself is far from having made up his mind as to the certainty of the disease ever having so very rapid a course.

In his views of the treatment of hypertrophy there is nothing particularly new. As to bloodletting, he lays it down as an approximative rule that it should be repeated three or four times, together with one or two cuppings of from eight to twelve ounces. In digitalis, (the true opium of the heart, as he terms it,) administered in the endermic manner, he seems to have much more confidence than most French practitioners.

*Atrophy of the Heart.* M. Bouillaud brings forward but a single case, that of a woman about sixty years of age, the beat of whose heart is described as having been extremely feeble, and very limited as to the extent in which it was perceptible to the hand. Its sounds were so weak as to be audible only in the intervals of the respiration: there was no bellows murmur. On dissection, the organ was found to be a full third less than the natural size, or about equal to that of a child of ten or twelve years old; wrinkled and withered, as it were, on the surface, where were observed some whitish patches, indicative of an old pericarditis. The left ventricle could scarcely contain a pigeon's egg, and the thickness of its walls did not exceed three lines; the cavity of the right ventricle was rather larger, and the walls were about one and a half lines thick. Burns has mentioned a much more marked case than this, where the heart of an adult did not exceed that of a new-born infant; and another, where the heart of a woman, of twenty-six years of age, was about equal in size to that of a child of six years. Amongst the local causes of this affection are enumerated the pressure of effused fluids within the pericardium, and contraction of the coronary arteries by disease; and, among constitutional causes, whatever tends to produce marasmus of the body at large, tubercular and cancerous diathesis, ulcerations of the intestines, &c., can scarcely fail to effect some alteration in the size of the heart.

The *Neuroses of the Heart* have met with little attention hitherto. Corvisart omits them altogether; Laennec dispatches them in a few pages; and Andral, in that part of his Medical Clinique treating of diseases of the heart, has treated them with as little ceremony. Our author commences this part of his subject by the consideration of nervous palpitations, which are so frequent in young persons addicted to excessive study and late hours, as well as in those who make an abuse of stimulants or indulge in sexual excesses; also in those of a chlorotic or anæmic habit, from whatever cause arising. The palpitations arising from the last-mentioned condition are daily confounded with organic diseases of the heart, to the infinite prejudice of the unfortunate patients; a mistake arising from the circumstance of chlorotic individuals being, as well as those labouring under organic affection, liable to dyspnœa and a sense of smothering from any exercise which is in the least fatiguing, as ascending a stair, &c. The diagnosis is the more important, as the treatment of the two diseases is diametrically opposite. This is a point to which we beg to call the most earnest attention of our readers, more especially our junior brethren, who are favourers of auscultation. We meet with no more common case of error in diagnosis and in practice than this, and we know of few more injurious, both to the system of the patient and the reputation of the practitioner. One half of the cases brought to men who have got a name for the treatment of diseases of the heart consist of these pseudo-cardiac affections. M. Bouillaud asserts that the chlorotic palpitations are for the most part unaccompanied by any well-marked bellows murmur in the heart itself; whilst, at the same time, this sound is almost constantly present in the great arteries, (carotids, crurals, &c.)

There is another variety of palpitations, which may be called the rheumatic, as they make their appearance in conjunction with wandering rheumatic pains about the præcordial region, darting from thence to the left arm: they are sometimes accompanied by slight intermittence of pulse, and cause considerable alarm to the sufferers, though in other respects they appear in high health. They are no more to be confounded with the palpitations originating in pericarditis or endocarditis of an acute rheumatic character, than is a pleurodynia with a pleurisy, being quite as distinct in their nature and tendency. Nervous palpitations are sometimes, no doubt, continuous; yet far more frequently they are, like most other nervous affections, intermittent. Andral and others are of opinion that their frequent recurrence may eventually give rise to organic disease, though there may have been nothing of this in the commencement; and, while in the stage of transition, the difficulty of making up one's mind as to the true nature of such cases is, it must be confessed, extremely difficult. By frequent examination with the stethoscope, especially in the absence of the palpitations, we must endeavour to ascertain whether the valves of the heart do their duty,—whether the orifices are in the natural condition, the walls hypertrophied or thinned,—as well as to satisfy ourselves, by the sight, touch, and percussion, whether there be enlargement of the organ. The absence of venous congestion, and of violet tint of the face, dropsies, &c., though the disease be already of some standing, adds to the probability of its being a mere nervous derangement. Yet, with all these aids, there are cases, as M. Bouillaud himself is obliged to confess, where there is very great difficulty in forming a correct opinion: namely, those complicated ones in which there exist simultaneously palpitations depending on organic disease and palpitations of a nervous nature; a kind of case more frequent, he believes, than has generally been suspected. M. Bouillaud insists earnestly on the importance of the physician using all his efforts to tranquillize his patient's mind, and relieve it from the vague apprehension of an organic disease.

With regard to *Neuralgia* of the heart, on which some stress has been laid by Laennec, M. B. is sceptical as to whether pains of the kind described have really their seat in this organ. That the pains are neuralgic, is unquestionable; but he thinks it more probable that they have their seat in the phrenic or intercostal nerves, and that in some cases it extends to the pneumogastric, cervical, and brachial plexus. Certain cases of nervous asthma, as well as of angina pectoris, he conceives may have this source. As to spasm of the heart, a disease likewise alluded to by Laennec, he believes it to be altogether an imaginary one, as there is nothing in the symptoms attributed to it indicative of true spasm. That this organ is sometimes liable to such an affection, in its more ordinary sense, is, however, we think, fully borne out by the group of morbid phænomena generally known under the name of Angina pectoris.

*Change of Dimensions in the Cavities.* In the section treating of changes in the dimension of the cavities and orifices of the heart, our author remarks that the contraction of the latter has been dwelt on by Corvisart and previous writers; but that the diminution of the cavities themselves had been quite overlooked, till Bertin pointed it out. Dilatation with thinning of the walls, or passive aneurism, as it was called in the old nomenclature, is much rarer than that accompanied with their thickening, or active aneurism. With regard to partial dilatation, or false consecutive aneurism of the heart, as it has been termed by M. Breschet, he remarks that, although insulated cases of it had from time to time been recorded, it is only lately that it has been studied with the minute detail which it merits. These aneurismal tumours are sometimes, but improperly, named *true* aneurisms of the heart: they are in reality, in almost every case, what we have called them above, *false consecutive* aneurisms; for the rupture of the interior layers of the parietes of the organ is one of their fundamental characters: but, as there is no rule without its exception, these ruptures have been, it is admitted, in some few cases, preceded by a partial dilatation. The situation in which this partial dilatation has been most frequently presented to M. Bouillaud's observation is the pulmonary portion of the right ventricle.

Dilatation of the orifices of the heart is no less frequent than that of its cavities, and very often accompanies the latter. The principal cause of both lies in obstacle to the circulation, and the consequent accumulation of blood and distention of the containing parts. The auricles are more prone to such dilatation than the ventricles, because their muscular parietes, being thinner, have less power of resistance; and for the same reason the right ventricle, when compared with the left, is as it were predisposed to it. The cavities immediately behind the obstruction are, generally speaking, the first to dilate; but this is not universally the case; for sometimes, in consequence of the unequal power of resistance of the different cavities, that which is nearest to the obstacle is not dilated so soon as one of the more distant ones. Thus, where there is induration of the aortic valves, with contraction of the orifice, the left auricle may become dilated before the left ventricle. Moreover, in consequence of the connexion and communication between the different cavities of the heart, any great obstruction to the course of the blood, wherever situated, may at length induce a general dilatation of all the cavities of this organ, and even of several of the larger vascular trunks. Amongst the more conspicuous exciting causes of dilatation of the heart are to be reckoned violent exercises, and all such occupations as make large demands on muscular exertion, passions of the mind, distortion of the vertebral column, and all the malformations tending to curtail the natural dimensions of the chest; and even, as our author subjoins, compression of the trunk by injudicious clothing. But, of all the causes, the pathological condition of the valves and orifices alluded to above is indubitably that which plays the chief part in its production.

We are under the necessity of passing over several chapters, partly because our limits will not allow us to notice them, but chiefly because they possess more of pathological than practical interest. All are, however, well worthy of perusal, and indeed demand the attentive study of every practitioner, who would be master of this important class of affections. The diseases which we omit are the following:—Syncope; Wounds of the Heart; Rupture of the Heart; Displacement of the Heart; Hernia of the Heart; Congenital Malformations of the Heart. The last class comprehends the following varieties: 1. dexiocardia, or transposition of the heart; 2. communication between the right and left cavities; 3. acardia, or absence of some of the parts constituting a normal heart; 4. bicardia, or increase of the number of normal parts; 5. malposition of the great vessels.

*Polypi of the Heart.* In an appendix, the oft-mooted question of the coagulation of the blood in the cavities of the heart, and the formation of polypiform emanations during life, is discussed. These polypi are divided into the recent or the amorphous, and those which have been formed a longer time before death, and give traces of incipient organization. Above twenty supposed cases of the latter are to be found in the course of the present work. As to the former, M. Bouillaud believes that the majority are found during or only shortly prior to the agonies of death, at the same time admitting that some may be of later origin; and that these are not very readily distinguishable from the others. When the coagula are colourless, very adherent, and closely twisted round the columnæ carneæ, there is reason to believe them of a date considerably anterior to death. Several of the additional cases of polypous concretions given in this appendix are taken from M. Legroux's work, published about eight or nine years ago, and little new light has we think been thrown on the subject since. M. L.'s account of their several stages is as follows:—first, the concretion forms, acquires a certain general density, and afterwards becomes softened in its centre, so as to form a kind of cyst filled with a sanious fluid, which subsequently assumes a purulent character; the pus is absorbed, the cyst becomes adherent to the adjacent parietes of the containing cavity, and penetrated by vascular ramifications. One of the best-marked instances of organized fibrine within the heart occurred to M. Senn, in the case of a girl of eighteen, in whom the right auricle was in great part filled by a concretion, in the centre of which there were vesicles full of a semi-concrete liquid. This polypiform concretion was traversed by an infinite number of vessels, some of a bright red, others of a dark colour. It extended into the superior cava, right subclavian and right jugular veins, &c. with the walls of which it seemed to be connected by a continuity of tissue. It is in the right side of the heart that these concretions most frequently occur, and the auricles are more commonly their seat than their respective ventricles. M. B. attributes the first of these circumstances to the course of the blood being more easily embarrassed in the right cavities, and partly also to the frequency of inflammation of the veins, and its propagation to the right side of the heart. He is inclined also to ascribe some influence to the greater disposition to coagulate in venous than in arterial blood. The anatomical character of these sanguineous concretions differs much, says M. Bouillaud, according to the period of their formation, and as they are mixed or not with pus or pseudo-membranous matter. The amorphous recent concretions do not differ essentially from the coagula formed in blood taken out of the body. The organized concretions, again, present very different appearances according to the date of their formation. In the first degree of organization, they are whitish, elastic, slightly adherent to the walls of the heart, and especially to the columnæ carneæ and tendons of the valves, round which they are often twisted. They are like the buffy coat formed on the surface of inflammatory blood, or like the false membranes of serous tissues in a state of incipient organization, and manifest various intermediate degrees of tenacity as they pass from the gelatinous to the fibrous condition. In a still more advanced state, they adhere by true cellular membrane to the parts on which they are formed, and into which they are as it were grafted, being penetrated by blood-vessels, and gradually acquiring additional firmness till they come to resemble in their structure fibrous polypi or fungous vegetations. He corroborates Legroux's account of their occasionally containing pus in their interior, so as to resemble cysts. They sometimes, as we have just seen, adhere firmly to the valves, and may thus lay the foundation of organic derangement to the form or structure of these important parts; but to the production of this effect the presence of inflammation would appear to be necessary. It is difficult to say whether these concretions are formed at the expense of the blood contained in the heart or are a secretion from the inflamed tissue. M. Legroux and M. Bouillaud incline to the latter opinion. The purulent matter occasionally contained in their interior is believed by the former of these gentlemen to be the product of their own secretion; but from this opinion our author dissents, believing that it comes rather from the lining membrane of the heart, or that it has been transported thither from some other part of the body, and has subsequently caused the formation of a clot in which it is at length enveloped; for it is found, even at that early period when the containing coagulum affords us yet little or no trace of organization.

The principal symptoms to which polypi of the heart can give rise are connected with the obstruction thrown in the way of the circulation by their occupation of the cavities and orifices of the organ. Their causes are either mechanical, viz. such as oppose the free current of the circulation, or chemico-vital.

The blood drawn from a patient in the agonies of death, or with great obstruction of the cardiac orifices, is very prone to coagulation, escaping from the vein in a thick semi-concrete state; and it is just in such cases that coagulation is most apt to take place within the heart, and give rise to those sudden and unexpected deaths so common in cases of heart disease. Of the chemico-vital causes of such coagulation, inflammation of the lining membrane of the heart is one of the principal; and the introduction of foreign substances into the torrent of the circulation, as pus, for example, in another. In the majority of the cases in the present work, where these fibrinous concretions were not connected with any mechanical obstacle to the circulation, they coincided either with an idiopathic inflammation of the heart or with an inflammation of some other organ reacting on the heart and entire circulation, the blood being then peculiarly prone to coagulate firmly, and form the buffy coat. These polypiform concretions, acting as obstacles to the circulation, and occurring especially in the right side of the heart, will necessarily, when in any considerable quantity, tend to impede the return of the blood from the different organs,—the brain, liver; or hence apoplectiform symptoms, serous effusions, &c.; and, as but a small quantity of the blood can reach the lungs, so as to be duly aerated, the phænomena of asphyxia will also occasionally manifest themselves. When their seat is in the left side, in addition to the above, dyspnœa will be a prominent symptom, inasmuch as the blood will be admitted with difficulty from the pulmonary veins, and congestion of the lung be thus effected. When the polypi interfere with the play of the valves, their effects will be the most distressing. The most remarkable of their symptoms are a tumultuous action of the heart, with dulness and diminution of the natural sounds; a bellows murmur, occasionally of a sibilant character; great anxiety; venous congestions followed by a comatose state and stertor, and occasionally preceded by convulsions, smallness of pulse, and coldness of the extremities. The sudden occurrence of such a group of morbid phænomena in the course of a pericarditis, or endocarditis, may sometimes enable us to announce their formation; and so also in chronic diseases of the heart, which had previously presented none of these violent symptoms. The prognosis in these cases is necessarily bad, and most so when the disease they accompany is in itself of a dangerous nature.—Treatment is almost out of the question.

In this article, which is rather an abstract than a review of M. Bouillaud's important work, we have endeavoured to place the actual state of knowledge in respect to cardiac disease before our readers, as fully as our limited space would admit of. We cannot take leave of the author without once more intimating that this, his greatest production, fulfils the promise of his earlier doings, and confers infinite credit on his zeal and industry. Like Morgagni's and some other standard books of former days, the one before us contains such a mass of carefully noted cases and dissections that whatever may be eventually the fate of the hypotheses in it,—(and they form after all, but a very inconsiderable portion of it,) or whatever future changes may take place in the theoretical parts of our art generally, this work must long keep its ground, and be held in high estimation as a rich magazine of well-observed pathological facts.

---

